# Computed tomography-guided aortic valve neocuspidization: details of preoperative assessment and surgical technique

**DOI:** 10.1093/icvts/ivac290

**Published:** 2023-01-09

**Authors:** Borys Todurov, Igor Mokryk, Bogdan Batsak, Nataliya Ponych

**Affiliations:** Department of Adult Cardiac Surgery, Heart Institute, Kyiv, Ukraine; Department of Adult Cardiac Surgery, Heart Institute, Kyiv, Ukraine; University Clinic of Taras Shevchenko National University, Kyiv, Ukraine; Department of Adult Cardiac Surgery, Heart Institute, Kyiv, Ukraine

**Keywords:** Aortic stenosis, Aortic valve neocuspidization, Ozaki procedure, Personalized treatment

## Abstract

The original Ozaki technique involves sizing and trimming the neovalve cusps during cross-clamp. It leads to prolongation of the ischaemic time, as compared to standard aortic valve replacement. We use preoperative computed tomography scanning of the patient's aortic root to develop personalized templates for each leaflet. With this method, autopericardial implants are prepared before the initiation of the bypass. It permits maximally adopting the procedure to the patient’s individual anatomy and to shorten the cross-clamp time. We present a case of a computed tomography-guided aortic valve neocuspidization and concomitant coronary artery bypass grafting with excellent short-term results. We discuss the feasibility and technical details of the novel technique.

## INTRODUCTION

Aortic valve neocuspidization (AVNeo) is becoming increasingly popular as a method of aortic valve replacement (AVR). Excellent short- and mid-term results are reported [1, 2]. An important disadvantage of the original AVNeo is the sizing and trimming of the leaflets during cross-clamp, leading to prolongation of the ischaemic time [3, 4]. Another drawback is that intercommissural distances are measured on the collapsed aorta. This may lead to incorrect sizing [3].

Over the last decades, cardiac computed tomography (CT) has become a valuable tool in guiding cardiovascular interventions [5]. The next frontier is translating the patient’s preoperative imaging data into custom-made devices designed for managing individual cases.

We present a method to develop personalized AVNeo leaflets based on preoperative CT scanning of the patient's aortic root (AR). With this technique, leaflets are measured in diastole and prepared before the initiation of the bypass. We used it for the surgical management of a patient with severe aortic stenosis and coronary artery disease.

## PATIENT AND METHODS

A 71-year-old male was referred to us for symptomatic aortic stenosis. Transthoracic echocardiography showed bicuspid aortic valve (AV) type I (R-L) with a peak gradient of 105 mmHg, a mean gradient of 69 mmHg; moderate aortic insufficiency; AV orifice area 0.7 cm^2^; aortic annulus diameter 22 mm; pulmonary artery pressure 45 mmHg; left ventricle ejection fraction 50%. Coronary angiography revealed three-vessel coronary artery disease (left anterior descending; obtuse marginal 1; posterior descending artery). The patient was qualified for surgical correction: CT-guided AVNeo and coronary artery bypass grafting (CABG).

Our goal was to create a competent AVNeo valve entirely based on preoperative CT measurements. An axial electrocardiogram-gated cardiac CT (GE OPTIMA CT660) was performed. To develop a realistic three-dimensional (3D) model of the patient’s AR data were exported to the 3D Slicer software (Version 4.11; http://www.slicer.org). The best AR image in diastole was selected. It was segmented based on an adjustable threshold. Only the voxels of interest (wall of the AR) were marked. Raphe between left and right coronary cusps was used as a guiding point for the commissure between the left and right AVNeo leaflets.

**Figure 1:**
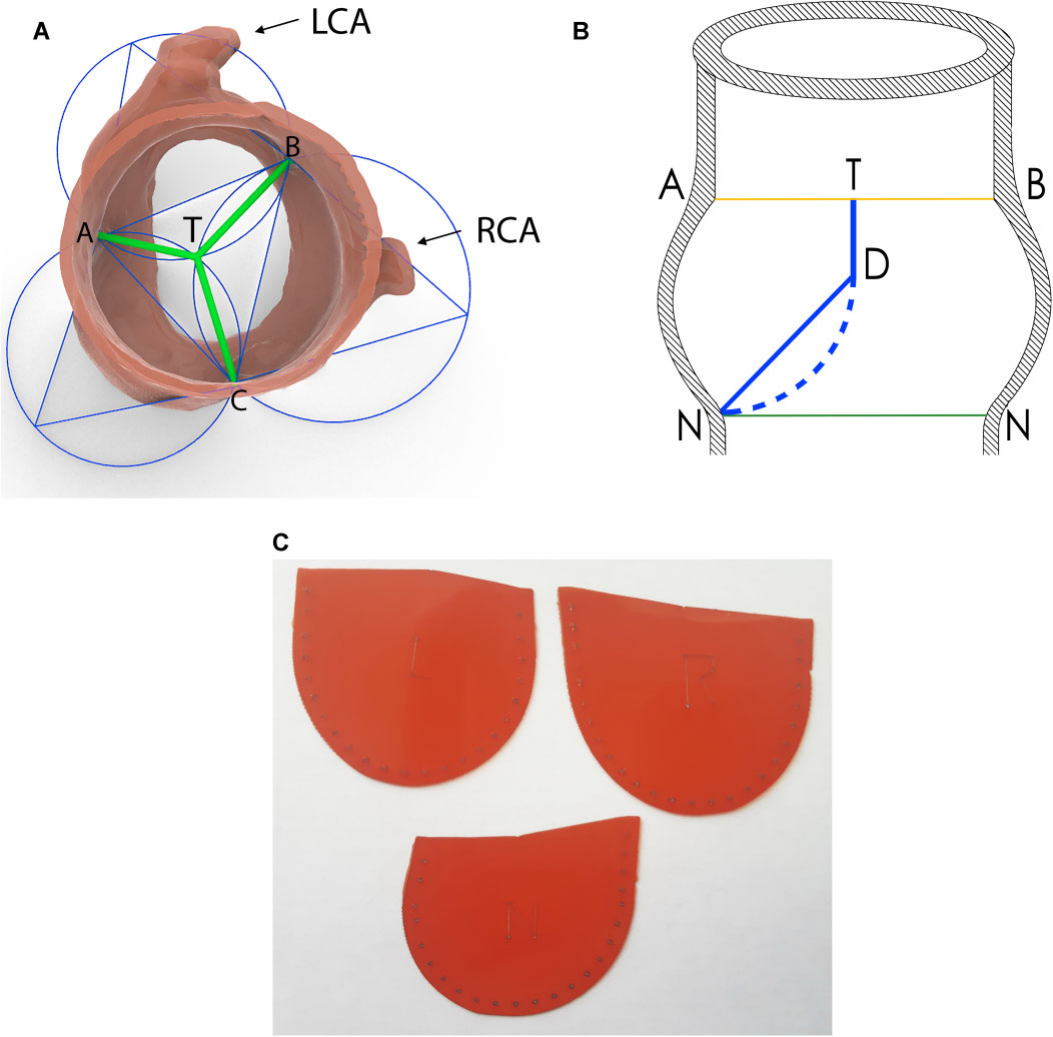
Development of the personalized AVNeo leaflets based on the preoperative 3D rendering of the patient’s aortic root. (**A**) Scheme of determining the length of the free margin of the patient-specific AVNeo leaflets. Localization of the Torricelli point (T) inside a triangle ABC, in which vertices are represented by the apexes of the commissures. The length of the free edge of the cusps is the sum of the segments from the vertices to the Torricelli point. Left coronary cusp = AT + BT; right coronary cusp = BT + CT; and non-coronary cusp = AT + CT. (**B**) Scheme to determine the height of the leaflet: cross-section of the AVNeo cusp through the plane of Torricelli and nadir points. Distance TD is the Coaptation Zone of the leaflet. Distance DN (solid line) multiplied by factor 1.1 is the belly part of the leaflet. Distance DN (dashed line) is the expected bulging of the leaflet after it is sutured down and distended under the blood pressure. (**C**) Personalized templates of computed tomography-guided AVNeo leaflets. A: the apex of the commissure between the left and non-coronary cusps; AVNeo: aortic valve neocuspidization; B: the apex of the commissure between the left and right coronary cusps; C: the apex of the commissure between the right and non-coronary cusps; D: caudal point of the coaptation zone; LCA: left coronary artery; N: the nadir; RCA: right coronary artery.

The concept of the reconstruction was to achieve all three cusps of the AVNeo valve coapting under 120°. The geometric solution to this problem was found by localization of the Torricelli point (TP) within the triangle, with vertices in the cranial points of the commissures (Fig. 1A). The length of the free edge of the cusp was calculated as the sum of the segments from the commissures to the TP. The height of the leaflet was determined from TP to the corresponding nadir. The coaptation zone (CZ) was designed to start at the horizontal plane of the commissures (Fig. 1B). The height of the CZ was set to be 8 mm. The distance from the caudal point of the CZ to the nadir was calculated using 3D Modelling Software and multiplied by factor 1.1 to permit future bulging. Adding to this number the height of the CZ resulted in the total height of the leaflet. The length and the configuration of the aortic annulus determined the length and the configuration of the sewing margin of the respective leaflet. Based on these data, a 3D model of the AVNeo cusp was created using 3D Modelling Software. It was converted into a two-dimensional image. Dimensions along the sewing margin were enlarged by 3 mm to allow plication while suturing. Plastic templates of each cusp were prepared and sterilized (Fig. 1C).

## SURGICAL TECHNIQUE

After midline sternotomy, an 8 cm × 9 cm piece of glutaraldehyde-treated autopericardium was prepared as a standard for the Ozaki procedure [1]. Leaflets were cut using personalized templates before the initiation of the bypass (Video 1). The principle of implantation is similar to the original Ozaki technique [1]. CT-guided AVNeo leaflets are smaller than standard Ozaki ones, approximately by one-quarter. Plication while suturing should still be performed, but not as extensive as with the original AVNeo. Commissures were reinforced with three oval-shaped 6 mm × 3 mm × 1.5 mm polytetrafluoroethylene pledgets usually utilized for standard valve replacement (Video 2). The final evaluation was performed by transoesophageal echocardiography after weaning from the bypass. It demonstrated a symmetric AVNeo valve with a transvalvular gradient of 14/8 mmHg and a trace aortic insufficiency.

Each step of the concomitant CABG (left internal mammary artery-to-left anterior descending and saphenous vein grafts to obtuse marginal 1 and posterior descending artery) was performed in a standard manner.

## RESULTS

**Figure 2:**
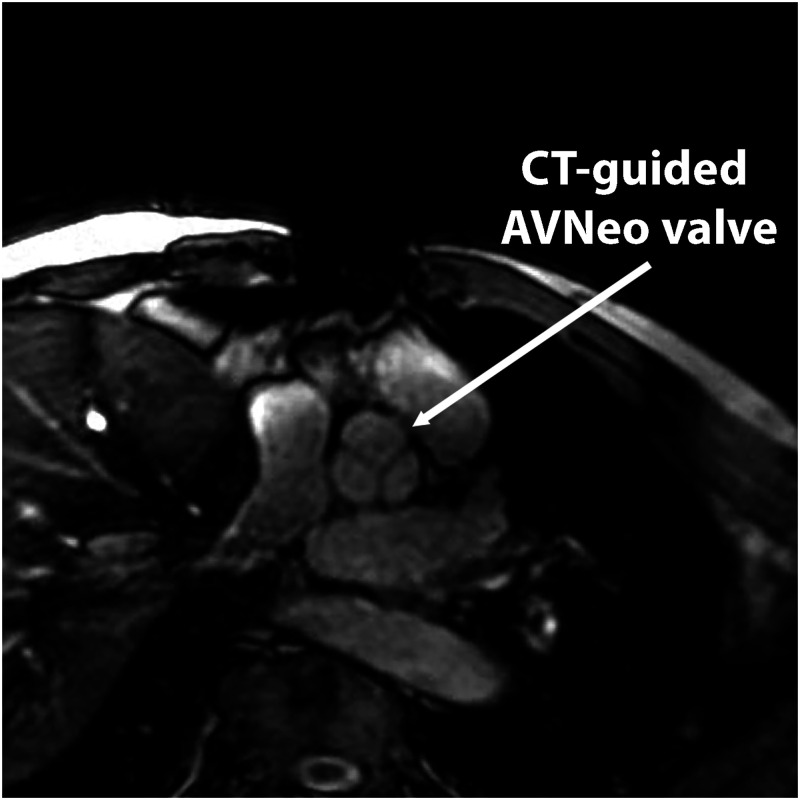
Cine-MR ‘on-valve plane’ view acquired 3 months postoperatively. The perfect geometry and function of the valve are demonstrated. AVNeo: aortic valve neocuspidization.

Aortic cross-clamp and bypass times were 108 and 151 min, respectively. The patient had an uneventful postoperative course. Intensive Care Unit stay was 2 days. The hospital stay after surgery was 7 days. Transthoracic echocardiography control at 1 month after discharge demonstrated the perfect function of the AVNeo valve with trace insufficiency and transvalvular gradient of 12/7 mmHg (Video 2). Magnetic resonance imaging control after 3 months showed excellent geometry of the AVNeo valve with good mobility and coaptation of the cusps (Fig. 2).

## DISCUSSION

The technical improvements in cardiac imaging methods over the last decades have led to the increasing popularity of 3D visualization (3DV) in interventional cardiology and cardiac surgery [5]. At the same time, data on the application of preoperative 3DV for the production of personalized devices to treat heart valve pathology are scarce [5].

The growing interest in the AVNeo procedure is based on a number of peculiar advantages of the method [1]. Preservation of left ventricle outflow tract mobility, large CZ, and absence of autoimmune response to glutaraldehyde-treated autopericardium are among the factors that resulted in excellent haemodynamic performance and a low complication rate of AVNeo up to the mid-term [1, 2]. At the same time, AVNeo is one of the most complex procedures in the surgery of the AV. Prolonged ischaemic time is an important disadvantage of the method [3, 4]. The preparation of the leaflets alone may constitute up to 20% of the ischaemic time [3]. Patients with combined cardiac pathology who require concomitant procedures constitute up to 43% of the cohort [1]. Reduction of the ischaemic time in this subgroup is especially important.

Another drawback of the original AVNeo is the measurement of the intercommissural distances on the collapsed aorta. The true dimensions, configuration and spatial relationships of the different structures of the AR may be estimated only when it is distended under physiologic blood pressure. During cross-clamp, all these characteristics significantly change. To mimic their actual size, the surgeon is recommended to put some pressure on the sizer while taking the measurement [1]. This manoeuvre is difficult to standardize and carries potential bias towards either over- or underestimated data.

There were previously reported experiences in the preoperative use of 3DV for planning and sizing AVNeo [3, 6, 7]. They demonstrated the feasibility of CT scanning for accurate preoperative procedure measurements.

We developed a method of CT-guided AVNeo permitting planning of the procedure and development of the patient-specific leaflets before the initiation of the bypass. Our method completely preserves the positive characteristics of the original Ozaki technique. At the same time, it has several essential advantages. A CT image of the configuration and size of the aortic annulus is obtained in the diastolic phase of the cardiac cycle when AVNeo leaflets will distend and close. This is an ideal environment for the surgical team to familiarize themselves with the unique patient anatomy, plan the procedure, and develop patient-specific AVNeo implants. They are closer in size to the native aortic leaflets than the original Ozaki ones. Their implantation requires lesser plication and may be performed faster. All these factors contribute to a reduction of the ischaemic and bypass time. As in our patient, they were 108 and 151 min, respectively. These data completely fall within standard limits for surgical AVR. Personalized templates provide an understanding of the size of the autopericardial flap sufficient to perform the procedure. This permits the prevention of the harvesting of an excessive amount of tissue. The three-pledget technique is a simple and fast method to reinforce neocommisures and ensure valve competence.

We consider CT-guided AVNeo as a step towards greater personalization of the Ozaki procedure. Our patient successfully underwent a CT-guided AVNeo with concomitant CABG. Excellent postoperative haemodynamic and clinical results prove the correctness of the preoperative measurements. However, we acknowledge that a greater number of clinical observations and longer follow-up are warranted to prove the viability of the concept.

**Conflict of interest:** none declared.

## Data Availability

This publication is based on our experience with one patient. The data underlying this article are available in the article and in its online supplementary material.

## Notes

Presented at the 36th EACTS Annual Meeting, Milan, Italy, 6 October 2022.
